# Nontoxic Multinodular Goitre and Incidental Thyroid Cancer: What Is the Best Surgical Strategy?—A Retrospective Study of 2032 Patients

**DOI:** 10.1155/2018/4735436

**Published:** 2018-05-14

**Authors:** Krzysztof Kaliszewski, Beata Wojtczak, Jędrzej Grzegrzółka, Jacob Bronowicki, Sawsan Saeid, Bartłomiej Knychalski, Zdzisław Forkasiewicz

**Affiliations:** ^1^First Department and Clinic of General, Gastroenterological and Endocrine Surgery, Wroclaw Medical University, Maria Sklodowska-Curie Street 66, 50-369 Wroclaw, Poland; ^2^Department of Human Morphology and Embryology, Division of Histology and Embryology, Medical University, Chalubinskiego Street 6a, 50-368 Wroclaw, Poland; ^3^Students' Scientific Club of the First Department and Clinic of General, Gastroenterological and Endocrine Surgery, Wroclaw Medical University, Wroclaw, Poland

## Abstract

**Objectives:**

A discussion with regard to the most optimal surgical procedure in nontoxic multinodular goitre (NTMNG). We assessed and compared three main types of operations in 2032 patients with NTMNG.

**Methods:**

This is a retrospective study of 2032 patients operated on in one center due to NTMNG. The observation period was 48 to 120 months (mean and SD: 87 ± 20).

**Results:**

The early complications included uni- and bilateral recurrent laryngeal nerve paralysis (URLNP, BRLNP), overt tetany (OT), and postoperative bleeding (POB). We observed after TT, STT, and DO URLNP: 15 (1.73%), 3 (0.64%), and 2 (0.28%), respectively (*p* < 0.05); BRLNP: 3 (0.34%), 2 (0.43%), and 0 (0.0%), respectively (*p* = 0.25); OT: 42 (4.84%), 6 (1.29%), and 9 (1.28%), respectively (*p* < 0.0001); and POB: 11 (1.26%), 4 (0.86%), and 3 (0.42%), respectively (*p* = 0.209). Persistent complications we observed after TT, STT, and DO are the following: URLNP: 9 (1.03%), 3 (0.64%), and 1 (0.14%), respectively (*p* = 0.086); BRLNP: 1 (0.11%), 1 (0.21%), and 0 (0.0%), respectively (*p* = 0.51); and OT: 11 (1.26%), 2 (0.43%), and 2 (0.28%), respectively (*p* = 0.052). Incidental thyroid cancer (ITC) was recognized after TT, STT, and DO in 18 (2.07%), 21 (4.52%), and 11 (1.56%), respectively (*p* = 0.039). Recurrent goitre (RG) was observed in 1 (0.11%) after TT, 3 (0.64%) after STT, and 2 (0.28%) after DO (*p* = 0.234).

**Conclusions:**

Performing less radical surgery in NTMNG is associated with a significantly lower risk of early and late URLNP and OT. In the case of BRLNP (early and persistent) and POB, no statistically significant differences are seen. The occurrence of ITC is higher following more radical surgeries. Less radical surgery is connected with a higher risk of RG.

## 1. Introduction

Surgical treatment has been regarded as an acceptable and appreciated method of nontoxic multinodular goitre (NTMNG) management [1]. The most common indications for surgery are compressive symptoms (dysphagia or shortness of breath), substernal extension, hyperthyroidism resistant to medical therapy, and suspicion of malignancy [1, 2]. Women are more likely to have thyroid disorders; however, in some studies, the overall prevalence of NTMNG does not differ between genders [3].

The choice of thyroid operation used in NTMNG (without suspicion of malignancy) is determined on an individual basis; however, the current guidelines and meta-analysis recommend total thyroidectomy (TT) for both toxic and NTMNG [4, 5]. The possible procedures include complete removal of both lobes of the thyroid (TT), subtotal removal of both lobes (subtotal thyroidectomy (STT)), complete removal of one lobe and subtotal the second (Dunhill operation (DO)), and adaptations of the aforementioned methods. A discussion is currently underway regarding the most optimal surgical procedure in treatment of NTMNG, without clinical and cytological features of malignancy. This is due to the multitude of surgical procedures that can be performed and a lack of clear, concise recommendations. In order to determine the most appropriate surgery for NTMNG, Albayrak et al. [6] analyzed in each surgical procedure the risk of early and late complications, ITC, and RG occurrence. They determined that when taking into account the rate of complications after reoperation due to RG and the risk of malignancy in RG, the most advantageous operation for NTMNG was TT. Other researchers also emphasized the higher rate of RG in less radical procedures [7] and consequently up to 20-fold higher increased risk of postoperative complications due to reoperation [8]. However, supporters of less radical procedures, particularly STT or DO, argued that these forms of treatment were associated with a lower risk of postoperative OT, as well as both URLNP and BRLNP [9, 10]. They further suggested that STT or DO might maintain thyroid hormone levels within the norm, eliminating the need for supplementation. Conversely, some authors concluded that “in the hands of experienced surgeons” TTs were equally as safe as less radical procedures [11]. They also argued that the risk of iatrogenic injury was comparable in both types of surgery (i.e., TT versus STT and DO).

Another widely discussed problem concerning the choice of surgical procedure in NTMNG is the rising occurrence of ITC [12]. Most thyroid nodules are benign; however, in 7–15% of cases, they are malignant [13]. Based on the analyzed literature and our own records, we noticed an increase of ITC diagnoses following management of NTMNG [14]. This observation is especially relevant in the case of NTMNG surgery. If a radical procedure is performed and ITC is diagnosed postoperatively, reoperation is not required. However, if a less radical procedure (e.g., STT or DO) is done, reoperation might be required and accompanied by an increased risk of postsurgical complications.

Owing to the numerous controversies surrounding the decision as to the choice of operation in NTMNG, it is worth to compare the three main types of operations in use—TT, STT, and DO. Within each procedure, it is valuable to evaluate early and late postoperative complications, the occurrence of ITC and RG after each procedure, as well as which procedures later require reoperation.

## 2. Materials and Methods

A retrospective analysis was performed on the medical records of 2032 patients operated on due to NTMNG in the First Department and Clinic of General, Gastroenterological and Endocrine Surgery in Wroclaw Medical University (Poland) from 2008 to 2013. Data were obtained from a clinical database created specifically for this study. Indications for surgical treatment were based on jointly accepted criteria used in the clinic, that is, compressive symptoms, progression in goitre size, and cosmetic reasons. A preoperative ultrasound-guided fine needle aspiration biopsy (UG-FNAB) of the dominant or selected tumor was performed in all patients in order to minimize the risk of malignancy occurrence in NTMNG. Exclusion criteria included suspicion of malignancy or follicular tumour (Bethesda stage ≥ 3) and hyperthyroidism (Graves' disease, toxic goitre), as these conditions could influence the choice of operation. Included in this analysis were patients with an established diagnosis of NTMNG (i.e., focal lesions in both lobes and euthyroidism), who also had UG-FNAB performed. Linear probes of 7.5–13.0 MHz were used. Three types of surgical procedures were identified: TT, STT, and DO. All operations were performed using similar surgical techniques by one team of endocrine surgeons specializing in thyroid surgery. In all surgeries, the similar approach, coagulation, ligation technique, equipment, and visualization of anatomical structures were used. A single dose of antibiotic was administered just before surgery in operating theatre sale. The surgeons intraoperatively tried to visualize all crucial anatomical structures like superior and recurrent laryngeal nerves and parathyroid glands, but not in all procedures, these goals were achieved. Regardless, there was no need for autotransplantation of accidentally removed parathyroid glands. All patients received hormonal supplementation with levothyroxine, in daily doses of 50–100 *μ*g depending on the choice of surgical procedure and body mass. The initial follow-up visit was six to eight weeks postthyroidectomy and the second visit six months after surgery. Patients were observed from a period of 48 to 120 months (mean and SD: 87 ± 20). All statistical analyses were performed using Prism 5.0 (GraphPad, La Jolla, USA). Chi-squared test was used. Results with a significance level *p* < 0.05 were considered statistically significant.

## 3. Results

**Figure 1:**
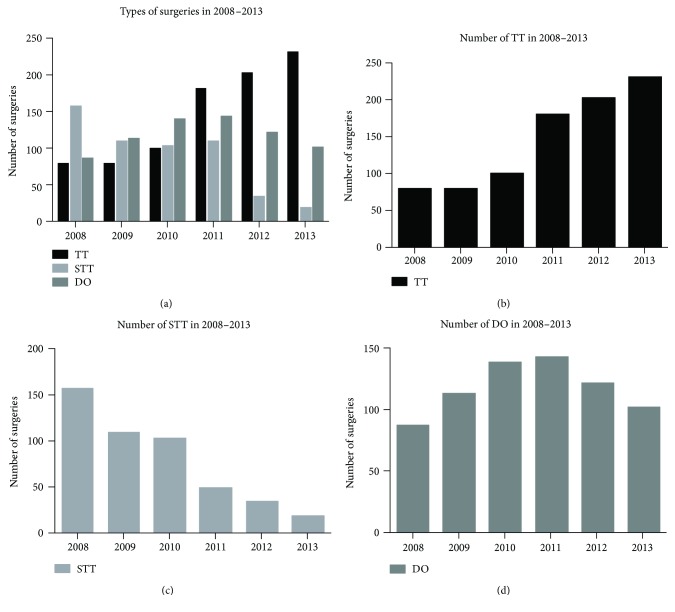
A constant increase in the number of more radical procedures (a, b) (total thyroidectomy (TT)) and a decrease in the number of less radical procedures (c, d) (subtotal thyroidectomy, STT, and Dunhill operation (DO)).

The percentages of early complications (URLNP, BRLNP, OT, and POB) in the successive analyzed years, per type of surgical procedure, are presented in Table 1. Postoperative bleeding requiring urgent revision of the surgical wound occurred following TT, STT, and DO, in 11 (1.26%), 4 (0.86%), and 3 (0.42%) cases, respectively. The overall postoperative bleeding accounts 0.88% (18/2032 patients). One patient after TT required repeated urgent revision due to a second postoperative hemorrhage. In the immediate postoperative period, there was no infection of the postoperative wound. In those patients who underwent radical treatment (TT), OT confirmed by hypocalcemia (levels less than 2.1 mmol/l) was observed in 42 (4.84%) individuals. The same complication occurred in 6 (1.29%) patients after STT and in 9 (1.28%) after DO, *p* < 0.0001 (Table 2). All patients who presented postoperatively OT (confirmed biochemically) received intravenous calcium preparations, then vitamin D3, and oral calcium supplementation. URLNP was observed in the following number of patients after TT, STT, and DO, respectively: 15 (1.73%), 3 (0.64%), and 2 (0.43%). BRLNP was observed in the following number of patients after TT, STT, and DO, respectively: 3 (0.34%), 2 (0.28%), and 0 (0.0%). However, only URLNP was statistically significant at *p* < 0.05 (Table 2). Persistent complications, evaluated six months postoperatively, were observed in the following number of patients after TT, STT, and DO, respectively: URLNP: 9 (1.03%), 3 (0.64%), and 1 (0.14%), *p* = 0.086; BRLNP: 1 (0.11%), 1 (0.21%), and 0 (0.0%), *p* = 0.51; and OT: 11 (1.26%), 2 (0.43%), and 2 (0.28%), *p* = 0.052 (Table 3). The incidence of ITC in NTMNG in the subsequent studied years (2008–2013) was 8 (2.3%), 4 (1.29%), 7 (1.96%), 5 (1.28%), 13 (2.98%), and 13 (2.75%), respectively. The occurrence of ITC after each type of surgery and for each year of the study is shown in Table 4. RG during the study period was observed in 1 (0.11%) patient after TT, in 3 (0.64%) after STT, and in 2 (0.28%) after DO (Table 4). It was not statistically significant (*p* = 0.234). In the patients who initially underwent less radical surgery (STT and DO), ITC was diagnosed in 32 (6.08%). They had to undergo a second operation in order to remove the remaining thyroid tissue. There was no need to reoperate the 18 (2.07%) individuals who underwent TT and in whom postoperative histopathology revealed malignancy, as they had received primary radical treatment, *p* = 0.039. All patients diagnosed with a malignant tumor after operation for NTMNG were routinely sent to the Oncology Center in Gliwice, for consultation and possible adjuvant therapy with radioiodine. During the course of our study (2008–2013), we observed a constant increase in the number of more radical procedures (TT) and a decrease in the number of less radical procedures (STT and DO) (Table 5, Figure 1). The number of TT increased from 24% in 2008 to 66% in 2013. The histopathological types and staging of ITC in MNG are presented in Table 6. No deaths occurred during observation period.

## 4. Discussion

There are several types of surgical procedures used in the treatment of NTMNG. Nonetheless, in recent years, there has been an increase in the use of radical surgeries, like total and near total thyroidectomy, and a decrease in the number of less radical ones (STT and DO). In our study, the percentage of radical surgeries (TT) increased from 24% in 2008 to 66% in 2013 (Figure 1). Each type of thyroid surgery, less or more radical, has advantages and disadvantages. Consequently, every decision regarding the choice of thyroidectomy should be made on an individual basis. Besides absolute medical indications for more radical treatment such as compressive symptoms, goitre progression, or suspicion of malignancy, other factors like age, concomitant disorders, and even patient expectation should be taken under consideration. Almost 20 years ego, some authors recommended more radical surgery in NTMNG [15]. They stressed that since the entire thyroid gland was affected, leaving behind even a small amount of tissue increased the risk of pathological changes, such as RG, necessitating reoperation. Other authors similarly agreed in that the main drawback of nonradical treatment was the greater risk of recurrence and a significantly higher risk of complications postsecondary treatment [16, 17]. Some studies found the recurrence rate after nonradical surgery in NTMNG to be very high, up to even 20% [7]. However, the recurrence rate described by various authors largely depended on the length of the postsurgical observation period. In the study by Rojdmark et al. [18], the observation period lasted 30 years and the rate of RG was 42%. On the other hand, Ozbas et al.'s [19] study had a shorter observation period of 2.5 years and a lower recurrence rate of 1.2% (after STT). In the meta-analysis conducted by Cao et al. [20] of seven major studies, it was found that RG after TT in NTMNG was lower than after STT or DO. Additionally, more radical procedures did not increase the risk of permanent complications. In our study, the difference in permanent postsurgical complications (URLNP, BRLNP, and OT) after radical and nonradical surgeries was lower than that in early complications, but still statistically significant (*p* = 0.005).

In opinion of the authors of this study, the problem of RG is more complex. RG in itself is not an issue. The problem arises when reoperation is needed. Thyroid reoperation is technically much more difficult and associated with higher risk of complications than the previous surgery. This is made by changed topography, altered anatomical situation, scar tissue development, and postsurgical adhesions. According to the literature, reoperation is associated with a ten times greater risk of recurrent laryngeal nerve injury and postsurgical OT, when compared to the initial operation [2, 21]. Authors correspondingly noticed that the risk of iatrogenic injuries directly correlated with the number of repeated operations.

Hormone supplementation is a topic frequently brought up by opponents of radical surgery [21]. They have advocated that less radical operations [STT or DO], which leave some thyroid tissue in situ, allow the patient to maintain a state of euthyroidism without the need for hormone supplementation. Opponents of less radical surgery quickly refuted this claim [22]. They proved that leaving behind thyroid tissue did not protect against hypothyroidism. According to their studies, 100% of patients after STT or DO required high doses of levothyroxine, up to 100 *μ*g a day. In our study, all patients regardless of the type of operation performed (TT, STT, or DO) received postoperative hormone supplementation in daily doses of 50–100 *μ*g (depending on the choice of surgical procedure and body mass). This dose was then adjusted, usually after 4–6 weeks, depending on the level of thyroid-stimulating hormone (TSH). During follow-up visits, none of the patients were able to discontinue hormone supplementation.

The incidence of thyroid cancer has increased dramatically over the last several decades [23], and thus the risk of ITC is an important component of the decision concerning the optimal operation in NTMNG. Its incidence in NTMNG is estimated at 4–18% [22, 24]. In such cases, performing a nonradical surgical procedure very often entails reoperation. The incidence of malignancy in a recurrent goitre ranged from 4% to 17% [25], and this closely correlated with the period of observation. The main advantages of TT are the radical removal of affected tissue, minimal risk of RG, and needless reoperation in the case of ITC found on postoperative histopathological examination [6, 14, 26, 27]. However, with total resection comes the potentially greater risk of postsurgical complications [22]. In our study, 18 (2.07%) patients after TT were diagnosed with ITC. Thus, performing a radical procedure meant these individuals could avoid reoperation. ITC was diagnosed after STT and DO, respectively, in 21 (4.52%) and 11 (1.56%) patients. Due to the initial nonradical approach, reoperation was required in these patients, and with it came a higher risk of postsurgical complications.

Some authors have proposed [7, 14, 26–28] that when the surgeon is trained in endocrine surgery, TT is equally as safe as less radical procedures. It was also recognized 24 years ago that TT carried out by a surgeon experienced in thyroid surgery was much safer when compared to less radical procedures performed by an inexperienced team [29]. Permanent URLNP after TT performed by an experienced team occurred in 0 to 0.7% of cases. Following STT performed by the same team, it occurred in 0 to 1.3% of all cases. Failure to intraoperatively localize the recurrent laryngeal nerve significantly increased the risk of URLNP and BRLNP [30].

During thyroidectomy, an extra effort should be made to preserve the blood supply to the parathyroid glands. Postoperative hypoparathyroidism can occur especially after more radical procedures and, consequently, clinically and laboratory evident hypocalcemia [31]. Some authors believed that the severity of OT was directly proportional to the extent of thyroidectomy [19]. However, the same authors reported that although early, transient OT depended on the extent of surgery, late and permanent OT showed no such dependence. They noticed a similar rate of permanent hypoparathyroidism after each type of operation, and the percentage of patients affected in TT, STT, and DO was 0.4%, 0.0%, and 0.0%, respectively. In this particular study, the majority of patients underwent DO with 1-2 g of thyroid tissue left behind. The authors proposed DO as a suitable alternative to all other types of thyroid surgery, as it combined the benefits of both total and subtotal thyroidectomies. It presented with a low RG rate and was associated with a low percentage of early, transient, and permanent OT. However, these authors agreed that if intraoperatively it was determined that a radical procedure could be carried out as safely as a less radical one, the former method should be performed. The idea behind this approach was to leave a minimal amount of thyroid tissue after surgical treatment, in case of the need for radioiodine treatment or completion of surgery due to ITC. As for DO, they did not observe a RG after this procedure, but it could be due to the very short period of observation. The authors concluded that surgeons experienced in endocrine surgery were free to perform radical surgery, because the type of procedure performed by them did not influence the incidence of complications. They also noted that the surgeon's experience had a greater impact on the incidence of complications after thyroidectomy than the type of procedure [19]. On the contrary, the others [32] determined that DO had no advantage over radical surgery. It differed from TT only by a slightly lower risk of hypothyroidism, which can be treated very well pharmacologically.

In our study, we estimated that performing less radical surgery in NTMNG was associated with a significantly lower risk of early and late URLNP and OT, than in more radical procedures. However, in the case of BRLNP (early and persistent) and POB, no statistically significant differences were seen. In addition, the occurrence of ITC was higher following a more radical surgery. Less radical surgery was connected with a higher risk of RG. A slightly growing trend in the occurrence of ITC in NTMNG over the study period was observed.

On the basis of the available literature, it seems that there is presently no conclusive evidence as to the superiority of one type of surgery over another in NTMNG. Additionally, it is impossible to unequivocally state whether radical or less radical surgical intervention should be the first-line treatment in NTMNG. Each patient should be carefully assessed, in consideration of the advantages and disadvantages of each type of operation. The aforementioned principle should be preserved in spite of the rising number of radical operations in recent years, at the expense of less radical procedures. In case of experienced endocrine surgeons, in a situation when it is intraoperatively clear that performing more radical surgeries would not increase the risk of complications in comparison to less radical surgery, the former approach may be performed.

The rising occurrence of ITC diagnosed after surgery for NTMNG could be another important justification for more radical procedures. Nonetheless, we think that in all patients with NTMNG without suspicion of malignancy, an individual approach should be applied.

## Figures and Tables

**Table 1:** The early complications in the successive analyzed years per type of surgical procedure.

Year	Type of surgery	Number *n* (%)	RLNP		OT	POB
			Unilateral	Bilateral		
2008	TT	78 (100%)	1 (1.28%)	0 (0.0%)	3 (3.84%)	0 (0.0%)
	STT	156 (100%)	1 (0.64%)	1 (0.64%)	2 (1.28%)	1 (0.64%)
	DO	86 (100%)	0 (0.0%)	0 (0.0%)	1 (1.16%)	0 (0.0%)

2009	TT	78 (100%)	1 (1.28%)	0 (0.0%)	5 (6.41%)	0 (0.0%)
	STT	108 (100%)	1 (0.92%)	0 (0.0%)	1 (0.92%)	0 (0.0%)
	DO	113 (100%)	0 (0.0%)	0 (0.0%)	2 (1.76%)	0 (0.0%)

2010	TT	99 (100%)	1 (1.01%)	0 (0.0%)	2 (2.02%)	1^∗^ (1.01%)
	STT	102 (100%)	0 (0.0%)	1 (0.98%)	0 (0.0%)	0 (0.0%)
	DO	138 (100%)	0 (0.0%)	0 (0.0%)	0 (0.0%)	1 (0.72%)

2011	TT	180 (100%)	0 (0.0%)	1 (0.55%)	8 (4.44%)	0 (0.0%)
	STT	48 (100%)	0 (0.0%)	0 (0.0%)	1 (208%)	1 (2.08%)
	DO	142 (100%)	0 (0.0%)	0 (0.0%)	1 (0.55%)	2 (1.1%)

2012	TT	202 (100%)	8 (3.96%)	2 (0.99%)	14 (6.93%)	2 (0.99%)
	STT	33 (100%)	0 (0.0%)	0 (0.0%)	2 (6.06%)	1 (3.03%)
	DO	121 (100%)	1 (0.82%)	0 (0.0%)	3 (2.47%)	0 (0.0%)

2013	TT	230 (100%)	4 (1.73%)	0 (0.0%)	10 (4.34%)	8 (3.47%)
	STT	17 (100%)	1 (5.8%)	0 (0.0%)	0 (0.0%)	1 (5.8%)
	DO	101 (100%)	1 (0.99%)	0 (0.0%)	2 (1.98%)	0 (0.0%)

Total		2032	20 (0.98%)	5 (0.24%)	57 (2.80%)	18 (0.88%)

RLNP: recurrent laryngeal nerve paralysis; OT: overt tetany; POB: postoperative bleeding; TT: total thyroidectomy; STT: subtotal thyroidectomy; DO: Dunhill operation. ^∗^Double hemorrhage after surgery, twice reoperated.

**Table 2:** Early complications depending on the specific type of surgery.

	Type of surgery	Complications		Chi^2^ *p*	URLNP		*p*	BRLNP		*p*
		Yes	No							
RLNP	TT	18 (2.07%)	849		15 (1.73%)	852		3 (0.34%)	864	
	STT	5 (1.07%)	459	*<0.01*	3 (0.64%)	461	***<0.05***	2 (0.43%)	460	*0.25*
	DO	2 (0.28%)	699		2 (0.28%)	699		0 (0.0%)	701	

OT	TT	42 (4.84%)	825							
	STT	6 (1.29%)	458	*<0.0001*						
	DO	9 (1.28%)	692							

POB	TT	11 (1.26%)	856							
	STT	4 (0.86%)	460	*0.209*						
	DO	3 (0.42%)	698							

Total complications	TT	71 (8.18%)	796							
	STT	15 (3.23%)	449	*<0.0001*						
	DO	14 (1.99%)	687							

RLNP: recurrent laryngeal nerve paralysis; URLPN: unilateral recurrent laryngeal nerve paralysis; BRLNP: bilateral recurrent laryngeal nerve paralysis; OT: overt tetany; POB: postoperation bleeding; TT: total thyroidectomy; STT: subtotal thyroidectomy; DO: Dunhill operation.

**Table 3:** Persistent complications, evaluated six months postoperatively.

Type of surgery	Type of persistent complications							
	URLNP (Chi^2^*p*)		BRLNP (Chi^2^*p*)		OT (Chi^2^*p*)		Total (Chi^2^*p*)	
TT	9 (1.03%)	*0.086*	1 (0.11%)	*0.51*	11 (1.26%)	*0.052*	21 (2.42%)	*0.005*
STT	3 (0.64%)		1 (0.21%)		2 (0.43%)		6 (1.29%)	
DO	1 (0.14%)		0 (0.0%)		2 (0.28%)		3 (0.42%)	

TT: total thyroidectomy; STT: subtotal thyroidectomy; DO: Dunhill operation; URLNP: unilateral recurrent laryngeal nerve paralysis; BRLNP: bilateral recurrent laryngeal nerve paralysis; OT: overt tetany.

**Table 4:** The occurrence of ITC after each type of surgery and for each year of the study.

Type of surgery	ITC in NTMNG							Chi^2^*p*	RG	Chi^2^*p*
	Year								Period	
	2008	2009	2010	2011	2012	2013	2008–2013		2008–2013	
TT	1	2	0	2	7	6	18 (2.07%)	*0.039*	1 (0.11%)	*0.234*
STT	5	1	5	3	3	4	21 (4.52%)		3 (0.64%)	
DO	2	1	2	0	3	3	11 (1.56%)		2 (0.28%)	
Total *n* (%)	8 (2.3%)	4 (1.29%)	7 (1.96%)	5 (1.28%)	13 (2.98%)	13 (2.75%)	50 (2.46%)		6 (0.29%)	

ITC: incidental thyroid cancer; NTMNG: nontoxic multinodular goitre; RG: recurrent goitre; TT: total thyroidectomy; STT: subtotal thyroidectomy; DO: Dunhill operation.

**Table 5:** Types of surgical procedures during the course of the study (2008–2013).

Type of surgery	Year						Total
	2008	2009	2010	2011	2012	2013	
TT	78 (24.37%)	78 (26.08%)	99 (29.20%)	180 (48.64%)	202 (56.74%)	230 (66.09%)	867 (42.66%)
STT	156 (48.75%)	108 (36.12%)	102 (30.08%)	48 (12.97%)	33 (9.26%)	17 (4.88%)	464 (22.83%)
DO	86 (26.87%)	113 (37.79%)	138 (40.70%)	142 (38.37%)	121 (33.98%)	101 (29.02%)	701 (34.49%)
Total	320 (100%)	299 (100%)	339 (100%)	370 (100%)	356 (100%)	348 (100%)	2032 (100%)

TT: total thyroidectomy; STT: subtotal thyroidectomy; DO: Dunhill operation.

**Table 6:** Histopathology and staging of incidental thyroid carcinoma according to AJCC 2010 classification in patients with multinodular goitre.

Histopathology type and staging	Number *n* (%)
Histopathological type, *n* (%)	
Papillary thyroid carcinoma	47 (94%)
Classical variant	44 (88%)
Follicular variant	3 (6%)
Follicular thyroid carcinoma	2 (4%)
Undifferentiated thyroid carcinoma	1 (2%)
TNM classification 2010, *n* (%)	
pT1a	30 (60%)
pT1b	17 (34%)
pT2	3 (6%)
pT3	0
pT4a	0
pT4b	0
pT (m)	3 (6%)
pNx	0
pMx	0
pTNM staging according to AJCC 2010, *n* (%)	
I	47 (94%)
II	3 (6%)
III	0
IV	0

## Data Availability

The data used to support the findings of this study are available from the corresponding author upon request.
